# Fate of *p*-hydroxycinnamates and structural characteristics of residual hemicelluloses and lignin during alkaline-sulfite chemithermomechanical pretreatment of sugarcane bagasse

**DOI:** 10.1186/s13068-018-1155-3

**Published:** 2018-06-05

**Authors:** Felipe A. M. Reinoso, Jorge Rencoret, Ana Gutiérrez, Adriane M. F. Milagres, José C. del Río, André Ferraz

**Affiliations:** 1 Departamento de Biotecnologia, Escola de Engenharia de Lorena, Universidade de São Paulo, Lorena, SP 12602-810 Brazil; 2 Instituto de Recursos Naturales y Agrobiología de Sevilla, CSIC, Av. Reina Mercedes, 10, 41012 Seville, Spain

**Keywords:** Grasses, Sugarcane, Recalcitrance, *p*-Hydroxycinnamates, Lignin, Hemicelluloses, Pretreatment, Alkaline-sulfite, Biorefinery

## Abstract

**Background:**

Preparing multiple products from lignocellulosic biomass feedstock enhances the profit and sustainability of future biorefineries. Grasses are suitable feedstocks for biorefineries as they permit a variety of possible by-products due to their particular chemical characteristics and morphology. Elucidating the fate of *p*-hydroxycinnamates (ferulates—FAs and *p*-coumarates—*p*CAs) and major structural components during bioprocessing helps to discriminate the sources of recalcitrance in grasses and paves the way for the recovery of *p*-hydroxycinnamates, which have multiple applications. To address these subjects, we assessed sugarcane bagasse biorefining under alkaline-sulfite chemithermomechanical (AS-CTM) pretreatment and enzymatic saccharification.

**Results:**

The mass balances of the major bagasse components were combined with 2D-NMR structural evaluation of process solids to advance our understanding of sugarcane bagasse changes during biorefining. AS-CTM pretreatment provided a high yield and thoroughly digestible substrates. The pretreated material was depleted in acetyl groups, but retained 62 and 79% of the original lignin and xylan, respectively. Forty percent of the total FAs and *p*CAs were also retained in pretreated material. After pretreatment and enzymatic hydrolysis, the residual solids contained mostly lignin and ester-linked *p*CAs, with minor amounts of FAs and non-digested polysaccharides. Saponification of the residual solids, at a higher alkali load, cleaved all the ester linkages in the *p*CAs; nevertheless, a significant fraction of the *p*CAs remained attached to the saponified solids, probably to lignin, through 4-*O* ether-linkages.

**Conclusion:**

AS-CTM pretreatment provided soundly digestible substrates, which retain substantial amounts of xylans and lignin. Acetyl groups were depleted, but 40% of the total FAs and *p*CAs remained in pretreated material. Ester-linked *p*CAs detected in pretreated material also resisted to the enzymatic hydrolysis step. Only a more severe saponification reaction cleaved ester linkages of *p*CAs from residual solids; nevertheless, *p*CAs remained attached to the core lignin through 4-*O* ether-linkages, suggesting the occurrence of an alkali-stable fraction of *p*CAs in sugarcane bagasse.

**Electronic supplementary material:**

The online version of this article (10.1186/s13068-018-1155-3) contains supplementary material, which is available to authorized users.

## Background

Biorefining lignocellulosic biomass to produce fuel, chemicals and heat has been the target of extensive research and development during the last few decades. Due to the high costs of biomass processing via biochemical routes, including pretreatment and enzymatic saccharification steps, multiple-product biorefineries have been noted to enhance the profit and sustainability of the process [1, 2]. For biochemical routes of biomass processing, at least one pretreatment step is necessary to enhance access to the structural polysaccharides [3]. Elucidating the physico-chemical mechanisms that describe the changes in biomass structure during pretreatment can help to reinforce biorefinery designs producing multiple products from the original feedstock.

Given their long-term use in the pulp and paper industry, pulping processes have served as the base for pretreatment in wood chips biorefining [4–6]. Sulfite chemithermomechanical (CTM) pulping is one of the industrially developed processes [7, 8] that have been successfully tuned for wood pretreatment [6]. However, lignocellulose from grasses, a relevant raw material for large biorefineries [9], differs from wood feedstocks concerning morphology and chemical composition and thus requires tailored investigations [10]. Grass internode morphology includes epithelial cells, and vascular bundles, which are rich in fibers and vessels, as well as large areas that are occupied by parenchymal cells [11, 12]. Nodes are even more complex and have been reported to be extremely recalcitrant [13]. The singular morphology of grasses makes them very heterogeneous material for pretreatment purposes.

From a chemical point of view, in addition to cellulose, hemicelluloses and lignin, *p*-hydroxycinnamates (ferulates and *p*-coumarates) comprise a significant portion of the aromatic compounds present in grass cell walls [14–16]. Ferulates (FAs) and *p*-coumarates (*p*CAs) form esters predominantly with hemicelluloses and lignin, respectively [16, 18], which contribute to cell wall recalcitrance [16]. Indeed, part of the *p*-hydroxycinnamates is detected as lignin via routine analytical techniques [14, 19].

In sugarcane, a detailed study discriminated lignin and *p*-hydroxycinnamates as core lignin and non-core lignin, respectively [19]. The authors suggested that approximately 60% of the original aromatics in sugarcane are non-core lignin based on the relative abundances of pyrolysis products detected by gas chromatography coupled with mass spectrometry. Non-core lignin was the first aromatic fraction to be released during the alkaline treatment of sugarcane bagasse, providing a less recalcitrant pretreated material, whereas core-lignin was shown to be more resistant [19]. UV microspectrophotometry also indicated that some of the *p*-hydroxycinnamates present in the fiber cell walls of sugarcane bagasse are resistant to alkaline-sulfite pretreatment [10]. Another study using rice straw treatment under mild alkaline conditions revealed that ester-linked FAs were promptly released into the treatment liquor, whereas ester-linked *p*CAs decreased simultaneously with lignin at a slower rate [20]. Under more severe reaction conditions, the 2D-NMR evaluation of sugarcane bagasse lignins recovered from soda/anthraquinone pulping liquors presented *p*CAs with “free” carboxylic acids linked to the core lignin through 4-*O* ether-linkages, suggesting the occurrence of an alkali-resistant *p*CA fraction in sugarcane [21].

Available data from the alkaline treatment of grasses suggest that some of the *p*-hydroxycinnamates remain attached to the pretreated material. Elucidating the fate of *p*-hydroxycinnamates and the structural characteristics of the residual lignin and xylan under these pretreatment conditions could help to discriminate sources of recalcitrance in sugarcane, which would enable new biorefinery models that include the recovery of *p*-hydroxycinnamates from grasses for multiple applications [22]. These subjects were assessed in the current work using alkaline-sulfite CTM (AS-CTM) pretreatment of sugarcane bagasse under increasing loads of chemicals to provide a set of samples suitable for showing progressive changes in the structural characteristics of the pretreated materials. The enzymatic saccharification, chemical composition and mass balance of the main bagasse components were combined with a detailed structural evaluation of the pretreated materials based on 2D-NMR experiments to advance understanding of sugarcane bagasse changes that occur during AS-CTM pretreatment.

## Methods

### Sugarcane bagasse and pretreatments

Sugarcane bagasse was obtained from a local mill, and it was air dried and stored under dry conditions. Table 1 includes the chemical composition of the studied sugarcane bagasse sample. The AS-CTM pretreatment was based on a previous report [23]. In the current study, sugarcane bagasse (air dried, non-milled material) was pre-cooked for 2 h at 120 °C using a solid:liquid ratio of 1:10 and three different alkaline-sulfite loads. In all experiments, the Na_2_SO_3_ load was twice that of NaOH and corresponded to 10, 5 or 2.5 g of Na_2_SO_3_ for each 100 g of dried sugarcane bagasse. Initial liquor pHs were 13.3, 13.0, and 12.7, respectively. Alkaline-sulfite pre-cooked sugarcane bagasse was separated from the cooking liquor by centrifugation, avoiding a washing step [24]. Retained solids were refined at 2.4% (w/v) solids in a disk refiner up to 250 Wh of energy consumption [23]. Pretreated material was recovered as a pulp slurry, which was dewatered by centrifugation up to a final humidity of 70%. Chlorite treatment was used to produce a reference sugarcane bagasse sample with reduced lignin content as previously described [25]. The reaction time was 2 h and the temperature was set to 70–80 °C.

**Table 1:** Chemical composition and mass balance for sugarcane bagasse components before and after pretreatment via an alkaline-sulfite chemithermomechanical process or chlorite delignification

Sample	Chemical composition of samples^a^ (g/100 g of pretreated sample)					Solids yield (%)	Mass balance (g/100 g of original sugarcane bagasse)				
	Lignin^b^	Glucan	Hemicellulose				Lignin	Glucan	Hemicellulose		
			Xylan	Arabinosyl	Acetyl				Xylan	Arabinosyl	Acetyl
Untreated	22.4 ± 0.6	40.0 ± 0.1	21.4 ± 0.1	2.0 ± 0.1	3.6 ± 0.1	100	22.4	40.0	21.4	2.0	3.6
2.5% Na_2_SO_3_ + 1.25% NaOH	24.8 ± 0.3	42.3 ± 0.5	20.4 ± 0.2	1.8 ± 0.1	1.4 ± 0.3	86.8	21.5	36.7	17.7	1.6	1.2
5% Na_2_SO_3_ + 2.5% NaOH	23.7 ± 0.3	44.0 ± 0.2	19.6 ± 0.1	1.9 ± 0.1	1.1 ± 0.2	84.1	19.9	37.0	16.5	1.6	0.9
10% Na_2_SO_3_ + 5% NaOH	16.8 ± 0.5	48.3 ± 0.3	20.5 ± 0.3	2.3 ± 0.1	0.1 ± 0.1	81.8	13.8	39.5	16.8	1.9	0.1
Chlorite-delignification	11.5 ± 0.5	43.8 ± 0.5	25.1 ± 0.7	2.8 ± 0.1	2.4 ± 0.4	85.7	9.9	37.6	21.5	2.4	2.1

^a^Original ash content in untreated sugarcane bagasse was 2.8 ± 0.7%

^b^Total lignin contents were not corrected for ash

### Chemical characterization, sugarcane bagasse component mass balance and sample microscopic evaluation

Before chemical characterization, all samples were air dried and milled to pass through a 0.84-mm screen. Untreated samples were extracted with 95 vol% ethanol for 6 h in a Soxhlet apparatus to characterize the extractives. Extracted samples and pretreated samples were hydrolyzed with sulfuric acid to determine their major components (lignin, glucan, xylan, arabinosyl groups and acetyl groups) as described elsewhere [26]. Triplicate analyses were performed for each sample. The reported data correspond to the average values and their standard deviations. Ash content in the sugarcane bagasse sample was 2.8 ± 0.7%. Owing to this low ash content, acid insoluble ash present in klason lignin was not discounted in the total lignin contents reported in the text.

Ferulic and *p*-coumaric acids were determined after the alkaline treatment of milled samples at mild (1 M NaOH at 30 °C for 24 h) and severe (4 M NaOH at 170 °C for 2 h) reaction conditions as previously described [14]. Triplicate analyses were performed for each sample. The reported data correspond to the average values and their standard deviations.

Mass balances for the major bagasse components, as well as for the FAs and *p*CAs, were calculated based on the initial contents that were recorded for the untreated samples and the final contents that were calculated as the percent composition multiplied by the solids yield recorded for each treatment condition.

Microscopic evaluation of the untreated sugarcane bagasse was performed after the samples were fixed in epoxy resin, cut along the transversal axes using a Leica EM UC7 ultramicrotome (1 µm thick), stained with toluidine blue and evaluated under an Olympus BX53 microscope as previously described [27]. After AS-CTM pretreatment, fibrous pretreated material was suspended in water and visualized under an Olympus BX53 microscope. After enzymatic hydrolysis of the pretreated material, the residual solids were air dried and macerated in a mortar and pestle. Milled solids were suspended in water and visualized under an Olympus BX53 microscope.

### Enzymatic hydrolysis of the pretreated materials

Pretreated bagasse samples were air dried, milled to pass through a 0.84-mm screen, and hydrolyzed with a commercial cellulase blend, Cellic CTec2 (SAE0020, SIGMA). The FPU activity of the commercial preparation was determined as described elsewhere [28]. Pretreated samples were suspended in 50-mL Falcon tubes with 50 mM acetate buffer containing 0.01% sodium azide (w/v), pH 4.8, at 2% solids (w/v). The suspended samples were mixed with commercial enzymes to assure 10 FPU/g of substrate in a total reaction volume of 10 mL. Samples were taken after different hydrolysis periods, with up to 72 h of reaction time. Samples were boiled at 100 °C for 5 min to stop enzymatic activity. The glucose and xylose contents were determined by HPLC [27]. Triplicate hydrolysis experiments were performed for each sample. The reported data correspond to the average values and their standard deviations. Cellulose and xylan conversions were calculated, respectively, from the amounts of glucose and xylose released during the hydrolysis, and they are expressed as a percentage of the theoretical maximum based on the glucan and xylan content of the pretreated material. To determine the yield of residual solids after 72 h of enzymatic digestion, the solids were washed 3 times with water, air dried and weighed.

### Saponification reactions of the solids remaining after enzymatic hydrolysis

The residual solids formed after enzymatic hydrolysis were crushed in a mortar and pestle and submitted to saponification at 90 °C for 48 h. Each reaction was performed inside a 10-mL Falcon tube containing 50 mg of sample and 5 mL of 1 M NaOH. After completion, the reaction mixture was adjusted to pH 2.0 with 6 M HCl, and water was added to a final volume of 10 mL. The mixture was stored at 4 °C overnight and was then filtered through a 0.45-μm membrane and analyzed by HPLC to measure the ferulic and *p*-coumaric acid contents as previously described [14]. To determine the yield of solids that remained after saponification, one analysis set was performed for each sample as described before, but residual solids were washed 3 times with water, air dried, and weighed.

## 2D-NMR characterization of pretreated materials and residual solids

2D-NMR characterization of the samples was performed using procedures already described [21, 24]. Briefly, approximately 50 mg of each biomass sample was dissolved in 0.75 mL of DMSO-*d*_6_, and NMR spectra were recorded at 25 °C using a Bruker AVANCE III 500 MHz instrument equipped with a cryogenically cooled 5-mm TCI gradient probe with inverse geometry (proton coils closest to the sample). Heteronuclear single quantum coherence (HSQC) experiments used Bruker’s ‘hsqcetgpsisp2.2’ pulse program (adiabatic-pulsed version) with spectral widths of 5000 Hz (from 10 to 0 ppm) and 23,843 Hz (from 165 to 0 ppm) for the ^1^H- and ^13^C-dimensions, respectively. The central solvent peak was used as an internal reference (δ_C_ 39.5; δ_H_ 2.49). HSQC correlation signals were assigned by comparing the results with previous evaluations of sugarcane bagasse samples [18, 21]. The ^1^H/^13^C correlation signals of H_2,6_, G2 and S2,6 in the aromatic region of the spectra were used to estimate the content of the respective H-, G- and S-lignin units (relative to the content of amorphous carbohydrates, estimated from the signals of the anomeric carbons in xylose, glucose and 4-*O* methyl-α-d-glucuronic acid). The signals for *p*CA2,6 and FA2 were used to estimate the abundance of the different *p*-hydroxycinnamates. The Cα/Hα correlation signals of the β-*O*-4′ alkyl aryl ethers (Aα) and phenylcoumarans (Bα) in the aliphatic-oxygenated region of the spectra were used to estimate their relative abundances (as per 100 aromatic units), whereas the Cγ/Hγ correlation signals were used to estimate the relative abundance of the cinnamyl alcohol end-units (as per 100 aromatic units).

## Results and discussion

### Pretreatment and enzymatic digestibility of pretreated samples

AS-CTM pretreatment performed with increasing alkaline-sulfite loads provided a set of pretreated sugarcane bagasse samples to investigate the fate of *p*-hydroxycinnamates (FAs and *p*CAs) and the structural characteristics of the residual hemicelluloses and lignin. A chlorite-delignified bagasse sample served as a reference material in the chemical characterization procedures and enzymatic hydrolysis experiments (Table 1).

Sulfite pretreatment enhances the efficiency of the enzymatic conversion of polysaccharides in several lignocellulosic materials [6, 29, 30]. In the case of sugarcane biomass, we demonstrated that this pretreatment can provide readily digestible materials depending on the chemical load used in the pretreatment and on the sugarcane cultivar [31]. In the current work, sugarcane bagasse from the industrial production of sugar and ethanol was pretreated with increasing alkaline-sulfite loads to generate a single set of results in which the same sugarcane bagasse lot was pretreated, digested with highly active commercial enzymes [32] and chemically characterized in detail.

Enzymatic hydrolysis of the pretreated samples corroborated previous data [31], showing improved hydrolysis efficiency with increased chemical loads in the pretreatment (Additional file 1: Figure S1). The initial enzymatic hydrolysis rates and extent of polysaccharide conversion after 72 h of incubation are summarized in Table 2. The initial hydrolysis rates for xylan were lower than those for glucan, probably because xylo-oligosaccharides accumulate in the reaction medium and thus require the inclusion of hemicellulases for a better hydrolysis response [33]. At a longer reaction time (72 h), the xylan conversion efficiency increased significantly but was still lower than that of glucan conversion. The glucan and xylan conversions reached their maximal values after pretreatment with the highest alkaline-sulfite load (95.6 and 78.9%, respectively). These glucan and xylan conversion efficiencies were slightly higher than those of a previous report [31]. The use of a highly active enzymatic cocktail in the current work seemed to yield improved enzymatic hydrolysis efficiency [32]. The reference chlorite-delignified sample was completely digested by the commercial enzymes after 72 h of incubation (Additional file 1: Figure S1).

**Table 2:** Initial polysaccharide hydrolysis rates and final conversion efficiencies during the enzymatic digestion of sugarcane bagasse pretreated via an alkaline-sulfite chemithermomechanical process at increasing chemical loads

Sample	Initial hydrolysis rate (% h^−1^)		Polysaccharide conversion after 72 h of enzymatic digestion (%)	
	Glucan	Xylan	Glucan	Xylan
2.5% Na_2_SO_3_ + 1.25% NaOH	2.6 ± 0.2	1.2 ± 0.2	31 ± 1	22 ± 1
5% Na_2_SO_3_ + 2.5% NaOH	8.0 ± 0.8	3.3 ± 0.7	58 ± 4	43 ± 3
10% Na_2_SO_3_ + 5% NaOH	9.9 ± 0.7	6.0 ± 0.6	96 ± 1	79 ± 1
Chlorite-delignified reference	11.7 ± 0.6	5.8 ± 0.3	100 ± 2	100 ± 2

### Mass balance and structural modifications of sugarcane bagasse during AS-CTM pretreatment

The chemical composition of the pretreated materials and a mass balance of bagasse components confirmed that lignin removal was the main effect of the AS-CTM pretreatment. Limited washing of the pretreated solids avoided excessive dissolution of xylan, providing high solids yields after pretreatment (Table 1). The highest Na_2_SO_3_/NaOH load used in the current experiments provided 38% lignin removal and xylan losses were limited to 20.6%. Acetyl groups were also extensively hydrolyzed, reaching up to 97% removal at the highest alkaline-sulfite load. Glucans were almost completely preserved. Selective lignin removal is characteristic of alkaline- and neutral-sulfite processes performed with less than 50% delignification. Higher delignification levels usually cause the simultaneous loss of polysaccharides [7]. In this context, alkaline-sulfite processes are suitable for biomass pretreatment purposes because 40–50% delignification has been proven to be enough to decrease biomass recalcitrance in a significant manner, permitting efficient polysaccharide conversion by commercial enzymes [25, 34, 35]. In the current work, the 2 h chlorite-delignified sample demonstrated this assertion for sugarcane bagasse, as lignin removal was selective, yet achieved 56% (Table 1), providing a substrate readily digestible by commercial enzymes (Table 2 and Additional file 1: Figure S1).

During AS-CTM pretreatment of sugarcane bagasse and after acid-chlorite delignification, FAs and *p*CAs were progressively removed at increasing chemical loads (Table 3). In grasses, FA is generally esterified to hemicelluloses (acylating the *O*-5 position of arabinosyl residues in arabinoxylans) with possible 4-*O*-β and 8-β cross-linkages to the lignin backbone; conversely, *p*CA is mainly esterified to the γ-OH of the syringyl (S) lignin units, typically as a terminal group in the polymer [16, 17].

**Table 3:** Hydroxycinnamic acids detected in sugarcane bagasse before and after pretreatment via an alkaline-sulfite chemithermomechanical process or chlorite delignification

Sample	Hydroxycinnamic acids (g/100 g of pretreated bagasse)				Solids yield (%)	Mass balance (g/100 g of original bagasse)			
	Mild hydrolysis		Severe hydrolysis			Mild hydrolysis		Severe hydrolysis	
	Ferulic	*p*-Coumaric	Ferulic	*p*-Coumaric		Ferulic	*p*-Coumaric	Ferulic	*p*-Coumaric
Untreated	0.28 ± 0.03	1.14 ± 0.08	1.5 ± 0.1	3.7 ± 0.4	100	0.28	1.14	1.5	3.7
2.5% Na_2_SO_3_ + 1.25% NaOH	0.20 ± 0.03	1.1 ± 0.1	1.2 ± 0.4	4.4 ± 0.3	86.8	0.17	0.94	1.1	3.8
5% Na_2_SO_3_ + 2.5% NaOH	0.11 ± 0.01	0.80 ± 0.05	1.4 ± 0.1	2.7 ± 0.3	84.1	0.09	0.67	1.2	2.3
10% Na_2_SO_3_ + 5% NaOH	0.11 ± 0.01	0.11 ± 0.03	0.7 ± 0.1	1.9 ± 0.4	81.8	0.09	0.09	0.6	1.5
Chlorite-delignified sample	0.08 ± 0.01	nd	0.16 ± 0.01	0.02 ± 0.03	85.7	0.06	nd	0.13	0.02

*nd* not detectable

The fate of FA and *p*CA during sugarcane bagasse pretreatment was initially evaluated by analytical determinations using mild (1 M NaOH at 30 °C) and severe (4 M NaOH at 170 °C) reaction conditions. These procedures would account for alkali-labile and total amounts of *p*-hydroxycinnamates, respectively [14, 36, 37]. Alkali-labile FA intensely decreased with increasing chemical loads in the pretreatment, whereas labile *p*CA decreased more gradually (Table 3). The total *p*-hydroxycinnamate content decreased with increasing alkaline-sulfite loads in the pretreatment but were detectable at high concentrations even after the most severe pretreatment (Table 3). Mass balances for total *p*-hydroxycinnamates indicated that 40% of the original FA and *p*CA content remained in the sample pretreated at the highest alkaline-sulfite load. It is noteworthy that alkaline conditions were maintained up to the end of each pretreatment condition (the final pH values of the pretreatment liquors were, respectively, 8.0, 8.5 and 9.8 for the increasing chemical loads used in the pretreatments). Nonetheless, some of the *p*-hydroxycinnamates remained attached to the pretreated materials. These data indicate that a significant fraction of the *p*-hydroxycinnamates was inaccessible to the alkaline liquor and/or that their ester linkages were cleaved, but they remained linked to the residual lignin and/or carbohydrates through 4-*O* ether cross-linkages.

**Fig. 1:**
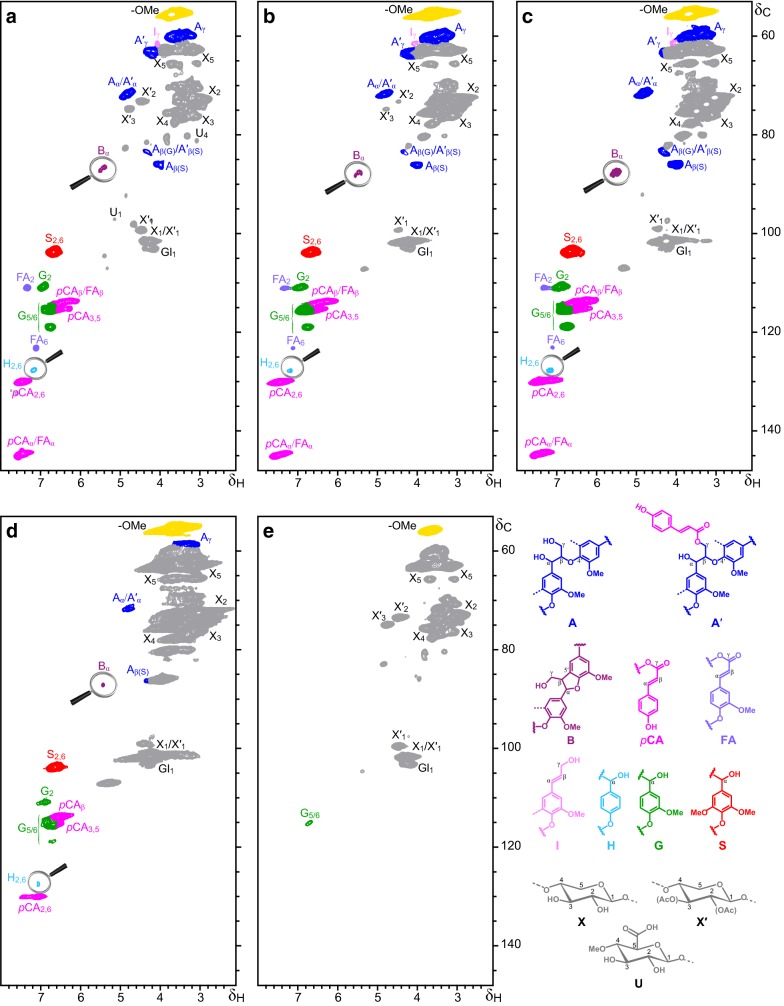
2D-NMR-HSQC spectra (δ_C_/δ_H_ 53–150/2.0–8.0) of **a** untreated sugarcane bagasse; **b** sugarcane bagasse pretreated with 2.5% Na_2_SO_3_ + 1.25% NaOH; **c** sugarcane bagasse pretreated with 5.0% Na_2_SO_3_ + 2.5% NaOH; **d** sugarcane bagasse pretreated with 10% Na_2_SO_3_ + 5.0% NaOH; and **e** reference-delignified sugarcane bagasse treated with sodium chlorite for 2 h. See Additional file 2: Table S1 for signal assignments. The main structures present in the sugarcane bagasse samples are **A**: β-*O*-4′ alkyl-aryl ethers; **A′**: γ-*p*-coumaroylated β-*O*-4′ alkyl-aryl ethers; **B**: β-5′ phenylcoumarans; **I**: cinnamyl alcohol end-groups; ***p*CA**: *p*-coumaric acid units; **FA**: ferulic acid units; **H**: *p*-hydroxyphenyl units; **G**: guaiacyl units; **S**: syringyl units; **X**: xylans; **X′**: acetylated xylans; and **U**: 4-*O* methyl-α-d-glucuronic acid. Note that the regions for C_α_/H_α_ correlation signal of phenylcoumarans (**B**_α_) and the H_2,6_ correlation signals of **H**-lignin units have been magnified for better viewing

2D-NMR-HSQC characterization of the pretreated samples clarified the fate of *p*-hydroxycinnamates as well as the structural characteristics of the residual hemicelluloses and lignin after pretreatment. Figure 1 presents the HSQC spectra of the untreated and AS-CTM pretreated sugarcane bagasse samples together with the chlorite-delignified reference sample. The main lignin and carbohydrate substructures that were found are also depicted in Fig. 1 and their correlation signals that were assigned in the HSQC spectra are listed in Additional file 2: Table S1. The HSQC spectra of the untreated and AS-CTM pretreated bagasse samples showed strong carbohydrate and lignin signals, whereas the spectrum of the reference-delignified sample essentially showed carbohydrate signals with a weak residual guaiacyl (G) lignin signal; the low concentrations of FA and *p*CA detected during wet chemical characterization of the chlorite-delignified sample (0.16 and 0.02%, respectively) were not detectable in the HSQC spectra. A semiquantitative analysis of the abundances of the different lignin units (H, G, S), *p*-hydroxycinnamates (*p*CA, FA), carbohydrates, and lignin inter-unit linkages and end groups was performed by volume integration of the respective correlation signals in the HSQC spectra and is shown in Table 4.

**Table 4:** Semiquantitative 2D-NMR analysis (abundances of lignin units, *p*-hydroxycinnamates, and carbohydrates, as per total lignin and carbohydrate units, and abundance of main lignin inter-unit linkages and end-groups, as per 100 lignin units) of the untreated and AS-CTM pretreated sugarcane bagasse samples

	Untreated	2.5% Na_2_SO_3 _+ 1.25% NaOH	5% Na_2_SO_3 _+ 2.5 NaOH	10% Na_2_SO_3 _+ 5% NaOH	RES-sap
Sample composition^a^					
*p*-Hydroxyphenyl lignin units (**H**)	0.6	0.4	0.4	0.1	0.6
Guaiacyl lignin units (**G**)	8.7	6.5	6.5	2.2	15.6
Syringyl lignin units (**S**)	10.1	8.1	7.5	2.3	13.4
**S**/**G** ratios	1.2	1.2	1.2	1.0	0.9
Total lignin units	19.4	15.0	14.4	4.6	ne
Total carbohydrate units	80.6	85.0	85.6	95.1	ne
*p*-Coumarates (***p*CA**)	10.4	10.0	9.0	1.7	2.5^c^
Ferulates (**FA**)	4.6	3.1	1.9	0.0	0.0
Main lignin inter-unit linkages and end-groups^b^					
β-*O*-4**′** Alkyl-aryl ethers (**A**)	60	59	49	42	4
Phenylcoumarans (**B**)	5	4	4	4	4
Cinnamyl alcohol end-groups (**I**)	3	3	3	0	2

The data for the residual solids that remained after enzymatic hydrolysis of the sugarcane bagasse sample pretreated with 10% Na_2_SO_3_ + 5% NaOH, and after extensive saponification (1 M NaOH, 48 h, 90 °C) (RES-sap) are also shown

*ne* not evaluated

^a^Sample composition represents the abundance of lignin units (**H**, **G**, **S**) and *p*-hydroxycinnamates (***p*CA**, **FA**) from integration of their respective signals, and carbohydrate units (xylose, glucose and 4-*O* methyl-α-d-glucuronic acid) from integration of the anomeric carbon signals, and are referred to the total lignin (**H** + **G** + **S**) and carbohydrate units (lignin + carbohydrate units = 100)

^b^The abundance of lignin linkages (**A**, **B**) and end-groups (**I**) is obtained from the integration of aliphatic signals and is referred to the total lignin units (**H** + **G** + **S** = 100)

^c^These represent the *p*CA that bear a free carboxylic group and are etherified at the 4-OH position to either lignin or carbohydrates

The most important hemicellulose signals in the spectra corresponded to the C2/H2, C3/H3, C4/H4, and C5/H5 correlations for xylans (X2, X3, X4, and X5) and the C4/H4 correlation for 4-*O* methyl-α-d-glucuronic acid (U4). Arabinosyl side groups were detected in the sugarcane samples only in low amounts (Table 1), but below the minimum needed to be detected in the HSQC spectra. Signals from *O*-acetylated xylans (3-*O*-acetyl-β-d-xylopyranoside, X′3, and 2-*O*-acetyl-β-d-xylopyranoside, X′2) were evident in the untreated sugarcane bagasse spectra. Other carbohydrate signals included the C1/H1 correlations for the anomeric carbons of β-d-xylopyranoside (X1), 2- and 3-*O*-acetyl-β-d-xylopyranosides (X′1), β-d-glucopyranoside (Gl1) and 4-*O* methyl-α-d-glucuronic acid (U1). All signal assignments for xylans confirmed that partially acetylated *O*-methylglucuronoxylans were the predominant hemicelluloses in sugarcane bagasse. Most of the carbohydrate signals observed in the HSQC spectra of the reference-delignified bagasse were also present in the spectra of the untreated bagasse and pretreated bagasse samples.

The signals from acetylated xylans (X′1, X′2 and X′3), which were present with substantial intensities in the spectra of the untreated bagasse and reference chlorite-delignified bagasse, gradually disappeared from the spectra of the pretreated bagasse samples as the alkaline-sulfite load increased (Fig. 1a–d). These data corroborated the wet chemical characterization results of the samples (Table 1), which indicated that the acetyl groups attached to the xylans were hydrolyzed and removed during the alkaline-sulfite pretreatment, but that part of the acetate was resistant to the treatment with up to 5% Na_2_SO_3_ and 2.5% NaOH (Fig. 1c). FA that usually esterifies arabinosyl side groups of hemicellulose in grasses [16] followed the same trend of the acetyl groups, as aromatic carbons from FA were detected with treatment at up to 5% Na_2_SO_3_ and 2.5% NaOH (Fig. 1c), corroborating the labile-FA contents determined using the mild hydrolysis procedure (Table 3). However, the residual total FA detected in the sample pretreated at the highest chemical load corresponded to 0.7% (Table 3), which was below the limit of detection of the HSQC (Fig. 1d, Table 4). U_1_ and U_4_ signals from *O*-methylglucuronic acid substituents were readily observed in HSQC spectra of the untreated sugarcane bagasse (Fig. 1a). After the mildest pretreatment (2.5% Na_2_SO_3_ and 1.25% NaOH), these signals were not detectable (Fig. 1b), suggesting that the *O*-methylglucuronic acid branched xylan fractions were preferentially solubilized during pretreatment.

Lignin signals were also observed in the HSQC spectra of the bagasse samples. The main lignin inter-unit linkages observed were β-*O*-4′ alkyl-aryl ethers (**A**). Intense signals in the δC/δH 62.7/3.83–4.30 range (which partially overlapped with the carbohydrate signals) from the Cγ/Hγ correlations of γ-acylated units demonstrated that *p*CA partially acylated the γ-OH in the lignin side-chains of some β-*O*-4′ substructures (**A′**) [18]. Detection of **A′** substructures by HSQC spectra corroborates the high contents of total *p*CA determined by wet chemical characterization (Table 3). Signals assigned to either C_β_/H_β_ or C_α_/H_α_ in β-*O*-4′ substructures (**A** and **A′**) decreased in intensity in the samples pretreated at the highest alkaline-sulfite loads, indicating that residual lignins were progressively depleted in β-*O*-4′ substructures (Table 4). Signals corresponding to phenylcoumaran structures (**B**) were also detected in the spectra and could be readily observed after magnification of the corresponding regions; contrary to β-*O*-4′ substructures, the abundance of phenylcoumarans remained essentially constant during the different treatments (Table 4). Signals corresponding to the Cγ/Hγ correlations of cinnamyl alcohol end groups (**I**) were also detectable in samples treated with up to 5% Na_2_SO_3_ and 2.5% NaOH (Fig. 1c, Table 4).

The main cross-signals in the aromatic regions of the HSQC spectra corresponded to the different lignin units and to the *p*-hydroxycinnamates. Signals from H-, G- and S-units were observed in the spectra of untreated (Fig. 1a) and AS-CTM pretreated samples (Fig. 1b–d). Although the total abundance of lignin units gradually decrease with alkaline-sulfite loads (Table 4), it appears that the S-units decrease more rapidly than H- and G-units with increasing alkaline-sulfite loads; this is particularly evident at the highest alkaline-sulfite loads, where the S/G ratios decrease from 1.2 to 1.0, reflecting the preferential removal of S-lignin units. This preferential removal is because S-lignin units are mostly involved in β-*O*-4′ ether linkages, which are more amenable to alkaline cleavage.

The HSQC data also corroborate that significant amounts of *p*CA remain in the pretreated samples, even after pretreatment at the highest alkaline-sulfite concentration. Characteristic signals from Cβ/Hβ correlations at δC/δH 113.5/6.27 were consistent with the esterified form of *p*CA in all bagasse samples, including the pretreated materials. The rest of the correlation signals observed in the spectra were also characteristic of *p*CA acylating the γ-OH in the lignin sidechain, as previously observed [18, 38]. From these data, it is possible to conclude that a part of the ester bonds between *p*CA and γ-OH in the lignin sidechain were resistant to the highest alkaline-sulfite load used in the pretreatment. The single explanation for preserved ester linkages after the alkaline-sulfite pretreatment under evaluation is limited accessibility to the *p*CA esters in the thick secondary cell walls present in sugarcane fibers [39]. Prompt alkali consumption by alkali-labile groups, such as free carboxylic acids, acetyl groups, phenolic structures in the extractives, phenolic lignin and the labile *p*-hydroxycinnamates, could compete with saponification of the less accessible *p*CA esters during pretreatment.

To advance structural information on the residual lignin in the pretreated samples as well as the fate of *p*CA under severe alkaline-sulfite pretreatment conditions, the sample pretreated with 10% Na_2_SO_3_ and 5% NaOH was hydrolyzed with commercial enzymes for 72 h at preparative scale (Additional file 3: Figure S2). Assaying the enzymatic hydrolysis liquid for FAs and *p*CAs resulted in non-detectable peaks using the HPLC procedure, suggesting that most of the *p*-hydroxycinnamates present in the pretreated material accumulated in the residual solids. Residual solids of the enzymatic hydrolysis procedure represented 16% of the initial mass of the pretreated sugarcane bagasse sample and mostly corresponded to lignin with *p*CAs as well as minor amounts of non-digested polysaccharides and FAs. The residual solids were further saponified at 90 °C, and they released only minimal amounts of *p*CAs and FAs to the reaction solution (10.8 and 4.8% of the total contained in the residual solids, respectively).

**Fig. 2:**
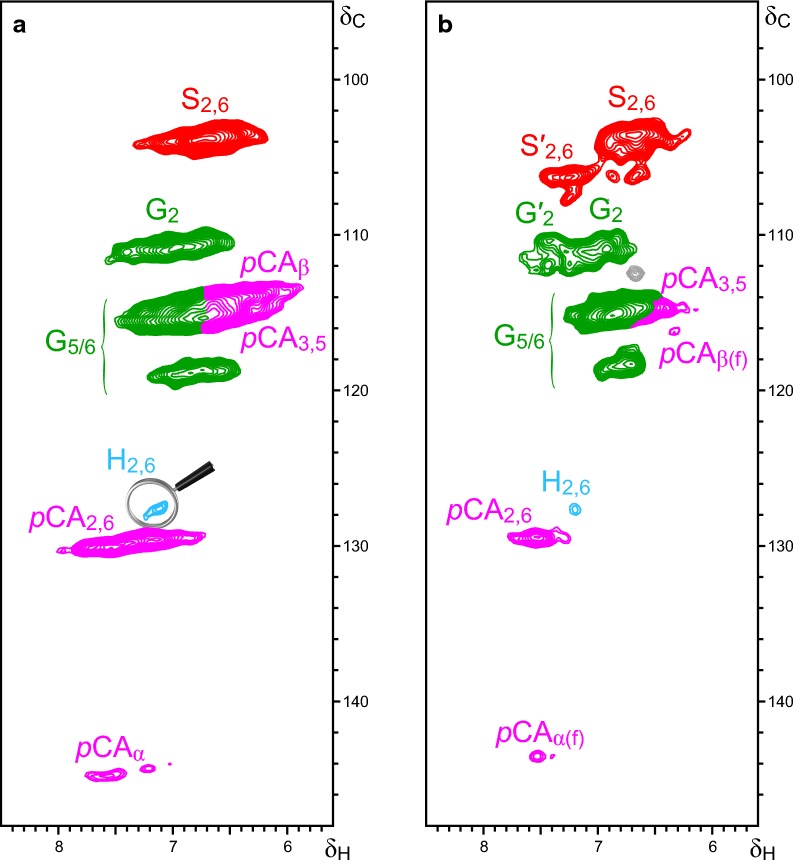
2D-NMR-HSQC spectra (δ_C_/δ_H_ 95–150/5.6–8.5) of **a** residual solids that remained after enzymatic hydrolysis of the sugarcane sample pretreated with 10% Na_2_SO_3_ + 5% NaOH and **b** the same residual solids after saponification conditions (1 M NaOH, 48 h, 90 °C). The main structures present in the sugarcane bagasse samples are **H**: *p*-hydroxyphenyl units, **G**: guaiacyl units; **G′**: Cα-oxidized G-units; **S**: syringyl units; **S′**: Cα-oxidized S-units; ***p*CA**: *p*-coumaric acid units; and ***p*CA**_**(f)**_: *p*CA units bearing a ‘free’ carboxylic group

The aromatic/unsaturated regions of the HSQC spectra of the residual solids (Fig. 2) helped to explain the fate of *p*CAs and FAs in the pretreated samples. Figure 2a corresponds to the spectrum of residual solids after enzymatic digestion of the polysaccharides. The C_β_/H_β_ and C_α_/H_α_ correlation signals of *p*CA esters were still observed, corroborating that the ester linkages of *p*CA acylating the lignin γ-OH were resistant to the most severe pretreatment as well as the enzymatic hydrolysis of the polysaccharide fraction. FA signals were not detectable due to their low amounts in the residual solids. After analytical saponification of the residual solids at 90 °C, the signals assigned to *p*CA esters were not detectable (Fig. 2b); instead, a characteristic shift of the C_β_/H_β_ signal to 116.3/6.32 ppm (*p*CA_β(f)_), together with a minor shift of the C_α_/H_α_ signal to 143.5/7.45 ppm (*p*CA_α(f)_), was observed and assigned to *p*CA units that bear a “free” carboxylic group, *p*CA_(f)_, but are etherified at the 4-OH position [21]. However, from the NMR data, it is not possible to conclude whether these *p*CA units are etherified to lignin or to carbohydrates. The abundances of the different lignin units (H, G, S), etherified *p*-hydroxycinnamic acids (*p*CA), carbohydrates, and lignin inter-unit linkages and end-groups in this sample (RES-sap), estimated from volume integration of the respective signals in the HSQC spectrum, are also shown in Table 4.

The compiled HSQC data demonstrate that all *p*CAs detected in the untreated sugarcane bagasse were originally γ-OH esterified to lignin. A fraction of these *p*CAs presented free OH groups at C-4 (representing mostly the labile *p*CA fraction released during pretreatment, which corresponded to 59% of the original amount—Table 3), whereas another fraction was also 4-*O* etherified to lignin and/or to carbohydrates at the same time (representing a less labile *p*CA fraction that was retained in the pretreated sample). Data from the residual solids after pretreatment and enzymatic hydrolysis indicated that the less labile *p*CAs retained ester linkages likely because the linkages were inaccessible to the alkaline-sulfite liquor owing to the complex cell wall structure of the biomass. After cell wall collapse due to polysaccharide dissolution (Additional file 3: Figure S2), these esters were promptly hydrolyzed by saponification reactions to *p*CAs bearing “free” carboxylic acids. However, the *p*CAs bearing “free” carboxylic acids remained linked to the residual lignin and or non-digested carbohydrates through 4-*O* ether-linkages.

Mass balances for total *p*CAs and FAs along treatments and analytical procedures are illustrated in Additional file 3: Figure S2. From 100 g of untreated sugarcane bagasse, the total *p*CAs and FAs corresponded to 3.7 and 1.5 g, respectively. After severe alkaline-sulfite pretreatment, labile *p*CAs and FAs were released into the pretreatment liquor, whereas the pretreated solids contained a residual amount of *p*-hydroxycinnamates corresponding to 1.5 and 0.6 g of *p*CAs and FAs, respectively. These amounts were still present in the residual solids used in the enzymatic hydrolysis procedure. When these residual solids were analytically saponified, 1.3 g of *p*CAs and 0.5 g of FAs remained attached to the solids, representing the less labile ether-linked *p*CAs and FAs.

## Conclusions

The current data strengthen evidence indicating AS-CTM pretreatment as a high-yield process that provides soundly digestible substrates, which can be converted to monosaccharides using a commercial cellulase cocktail. The pretreated material prepared at the highest alkaline-sulfite load retained high amounts of xylans that were depleted in acetyl groups. Part of the original lignin (62%) and 40% of the total FAs and *p*CAs were also retained in the pretreated material. The partial removal of lignin, xylans and *p*-hydroxycinnamates, and complete deacetylation (at the highest alkaline-sulfite load) proved to be sufficient to result in an almost completely digestible pretreated material. None of the isolated biomass modifications would improve digestibility to values as high as 80–95% for polysaccharide conversion. Combined transformations of the substrate after pretreatment were decisive for improving digestibility. Deacetylation, deferuloylation, and the preferential dissolution of *O*-methylglucurono-xylans should enhance the enzymatic hydrolysis of residual xylan, thus contributing to increased access to cellulose. Simultaneous lignin and *p*-hydroxycinnamates removal also increased cellulose accessibility. After pretreatment and enzymatic hydrolysis, residual solids contained mostly lignin and ester-linked *p*CAs, with minor amounts of FAs and non-digested polysaccharides. Saponification of the residual solids cleaved all the ester linkages in the *p*CAs; nevertheless, a significant fraction of *p*CAs remained attached to the core lignin and/or non-digested carbohydrates through 4-*O* ether-linkages.

Elucidating the fate of *p*-hydroxycinnamates and the structural characteristics of residual lignin and xylans under these pretreatment conditions helped to discriminate sources of recalcitrance in sugarcane and will inform new biorefinery models for grasses, which should include *p*-hydroxycinnamate recovery (from pretreatment liquor and residual lignin) for multiple applications.

## Additional files

**Additional file 1: Figure S1.** Time course of the enzymatic glucan and xylan conversion of sugarcane bagasse after chemithermomechanical pretreatment with increasing alkaline-sulfite loads. A reference chlorite-delignified sugarcane bagasse sample is also included in the dataset.

**Additional file 2: Table S1.** Assignments of ^1^H/^13^C correlation signals in the 2D HSQC spectra from untreated and pretreated sugarcane (*Saccharum* spp.) bagasse in DMSO-*d*_6_.

**Additional file 3: Figure S2.** Processes diagram, main process steps and transformations, and mass balances for sugarcane bagasse and total *p*CAs and FAs by treatment and analytical procedure. The photomicrography of the untreated sugarcane bagasse was obtained after toluidine blue staining.

## References

[1] Cheali P, Gernaey KV, Sin G (2014). Toward a computer-aided synthesis and design of biorefinery networks: data collection and management using a generic modeling approach. ACS Sustain Chem Eng.

[2] Budzianowski WM (2017). High-value low-volume bioproducts coupled to bioenergies with potential to enhance business development of sustainable biorefineries. Renew Sustain Energy Rev.

[3] Silveira MHL, Morais ARC, Lopes AMC, Olekszyszen DN, Bogel-Łukasik R, Andreaus J, Ramos LP (2015). Current pretreatment technologies for the development of cellulosic ethanol and biorefineries. Chem Sustain Energy Mater.

[4] Jin Y, Jameel H, Chang HM, Phillips R (2010). Green liquor pretreatment of mixed hardwood for ethanol production in a repurposed kraft pulp mill. J Wood Chem Technol.

[5] Qin Y, Yu L, Wu R, Yang D, Qiu X, Zhu JY (2016). Biorefinery lignosulfonates from sulfite-pretreated softwoods as dispersant for graphite. ACS Sustain Chem Eng.

[6] Zhou HF, Zhu JY, Gleisner R, Qiu XQ, Horn E, Negron J (2016). Pilot-scale demonstration of SPORL for bioconversion of lodgepole pine to bioethanol and lignosulfonate. Holzforschung.

[7] Kordsachia O, Fehr J, Csoka L, Winkler A (2008). ASA and kraft pulping of poplar. Cellul Chem Technol.

[8] Gellerstedt G, Ek M, Gellerstedt G, Henriksson G (2009). Chemistry of chemical pulping. Pulping chemistry and technology.

[9] Weijde T, Kamei CLA, Torres AF, Vermerris W, Dolstra O, Visser RGF, Trindade LM (2013). The potential of C4 grasses for cellulosic biofuel production. Front Plant Sci.

[10] Mendes FM, Fonseca MB, Ferraz A, Milagres AMF (2016). Anatomic and ultrastructural characteristics of different regions of sugar cane internodes which affect their response to alkaline-sulfite pretreatment and material recalcitrance. Energy Fuels.

[11] Moore PH, Heinz DJ (1987). Anatomy and morphology. Sugarcane improvement through breeding.

[12] Xue J, Zhao Y, Gou L, Shi Z, Yao M, Zhang W (2016). How high plant density of maize affects basal internode development and strength formation. Crop Sci.

[13] Brienzo M, Abud Y, Ferreira S, Corrales RCNR, Ferreira-Leitao VS, de Souza W, Sant’Anna C (2016). Characterization of anatomy, lignin distribution, and response to pretreatments of sugarcane culm node and internode. Ind Crops Prod.

[14] Masarin F, Gurpilhares DB, Baffa DCF, Barbosa MHP, Carvalho W, Ferraz A, Milagres AMF (2011). Chemical composition and enzymatic digestibility of sugarcane selected for varied lignin content. Biotechnol Biofuels.

[15] Siqueira G, Milagres AMF, Carvalho W, Koch G, Ferraz A (2011). Topochemical distribution of lignin and hydroxycinnamic acids in sugar-cane cell walls and its correlation with the enzymatic hydrolysis of polysaccharides. Biotechnol Biofuels.

[16] Hatfield RD, Rancour DM, Marita JM (2017). Grass cell walls: a story of cross-linking. Front Plant Sci.

[17] Ralph J, Hatfield RD, Quideau S, Helm RF, Grabber JH, Jug HJG (1994). Pathway of *p*-coumaric acid incorporation into maize lignin as revealed by NMR. J Am Chem Soc.

[18] del Río JC, Lino AG, Colodette JL, Lima CF, Gutiérrez A, Martínez AT, Lu F, Ralph J, Rencoret J (2015). Differences in the chemical structure of the lignins from sugarcane bagasse and straw. Biomass Bioenergy.

[19] Martínez PM, Punt AM, Kabel MA, Gruppen H (2016). Deconstruction of lignin linked *p*-coumarates, ferulates and xylan by NaOH enhances the enzymatic conversion of glucan. Bioresour Technol.

[20] Linh TN, Fujita H, Sakoda A (2017). Release kinetics of esterified *p*-coumaric acid and ferulic acid from rice straw in mild alkaline solution. Bioresour Technol.

[21] Menezes FF, Rencoret R, Nakanishi SC, Nascimento VM, Silva VFN, Gutiérrez A, del Río JC, Rocha GJM (2017). Alkaline pretreatment severity leads to different lignin applications in sugar cane biorefineries. ACS Sustain Chem Eng.

[22] Taofiq O, González-Paramás AM, Barreiro MF, Ferreira ICFR (2017). Hydroxycinnamic acids and their derivatives: cosmeceutical significance, challenges and future perspectives, a review. Molecules.

[23] Mendes FM, Siqueira G, Carvalho W, Ferraz A, Milagres AMF (2011). Enzymatic hydrolysis of chemithermomechanical pretreated sugarcane bagasse and samples with reduced initial lignin content. Biotechnol Progr.

[24] Sporck D, Reinoso FAM, Rencoret J, Gutiérrez A, del Río JC, Ferraz A, Milagres AMF (2017). Xylan extraction from pretreated sugarcane bagasse using alkaline and enzymatic approaches. Biotechnol Biofuels.

[25] Siqueira G, Várnai A, Ferraz A, Milagres AMF (2013). Enhancement of cellulose hydrolysis in sugarcane bagasse by the selective removal of lignin with sodium chlorite. Appl Energy.

[26] Ferraz A, Baeza J, Rodriguez J, Freer J (2000). Estimating the chemical composition of biodegraded pine and eucalyptus wood by DRIFT spectroscopy and multivariate analysis. Bioresour Technol.

[27] Costa THF, Masarin F, Bonifácio TO, Milagres AMF, Ferraz A (2013). The enzymatic recalcitrance of internodes of sugar cane hybrids with contrasting lignin contents. Ind Crops Prod.

[28] Ghose TK (1987). Measurement of cellulase activities. Pure Appl Chem.

[29] Leu S-Y, Zhu JY (2013). Substrate-related factors affecting enzymatic saccharification of lignocelluloses: our recent understanding. Bioenergy Res.

[30] Liu ZJ, Lan TQ, Li H, Gao X, Zhang H (2017). Effect of bisulfite treatment on composition, structure, enzymatic hydrolysis and cellulase adsorption profiles of sugarcane bagasse. Bioresour Technol.

[31] Laurito-Friend DF, Mendes FM, Reinoso FM, Ferraz A, Milagres AMF (2015). Sugarcane hybrids with original low lignin contents and high field productivity are useful to reach high glucose yields from bagasse. Biomass Bioenergy.

[32] Rodrigues AC, Haven MØ, Lindedam J, Felby C, Gama M (2015). Celluclast and Cellic^®^CTec2: saccharification/fermentation of wheat straw, solid–liquid partition and potential of enzyme recycling by alkaline washing. Enzyme Microb Technol.

[33] Sun FF, Hong J, Hu J, Saddler JN, Fang X, Zhang Z, Shen S (2015). Accessory enzymes influence cellulase hydrolysis of the model substrate and the realistic lignocellulosic biomass. Enzyme Microb Technol.

[34] Lee SH, Doherty TV, Linhardt RJ, Dordick JS (2009). Ionic liquid-mediated selective extraction of lignin from wood leading to enhanced enzymatic cellulose hydrolysis. Biotechnol Bioeng.

[35] Tian D, Chandra RP, Lee JS, Lu C, Saddler JN (2017). A comparison of various lignin-extraction methods to enhance the accessibility and ease of enzymatic hydrolysis of the cellulosic component of steam-pretreated poplar. Biotechnol Biofuels.

[36] Lam TBT, Iiyama K, Stone BA (1994). Determination of etherified hydroxycinnamic acids in cell walls of grasses. Phytochem.

[37] Grabber JH, Ralph J, Hatfield RD (2000). Cross-linking of maize walls by ferulate dimerization and incorporation into lignin. J Agric Food Chem.

[38] Poelking VGC, Giordano A, Ricci-Silva ME, Williams TCR, Peçanha DA, Ventrella MC, Rencoret J, Ralph J, Barbosa MHP, Loureiro M (2015). Analysis of a modern hybrid and an ancient sugarcane implicates a complex interplay of factors in affecting recalcitrance to cellulosic ethanol production. PLoS ONE.

[39] SanJuan R, Anzaldo J, Vargas J, Turrado J, Patt R (2001). Morphological and chemical composition of pith and fibers from mexican sugar cane bagasse. HolzAlsRoh-Und Werkstoffholz.

