# Supplementary material for: Fate of p-hydroxycinnamates and structural characteristics of residual hemicelluloses and lignin during alkaline-sulfite chemithermomechanical pretreatment of sugarcane bagasse
Source: Biotechnol Biofuels. 2018 Jun 5;11:153. doi: 10.1186/s13068-018-1155-3 (PMC5987574; doi:10.1186/s13068-018-1155-3)
Supplement: Supplementary file 1 — Additional file 1: Figure S1. Time course of the enzymatic glucan and xylan conversion of sugarcane bagasse after chemithermomechanical pretreatment with increasing alkaline-sulfite loads. A reference chlorite-delignified sugarcane bagasse sample is also included in the dataset. [file 13068_2018_1155_MOESM1_ESM.pdf]

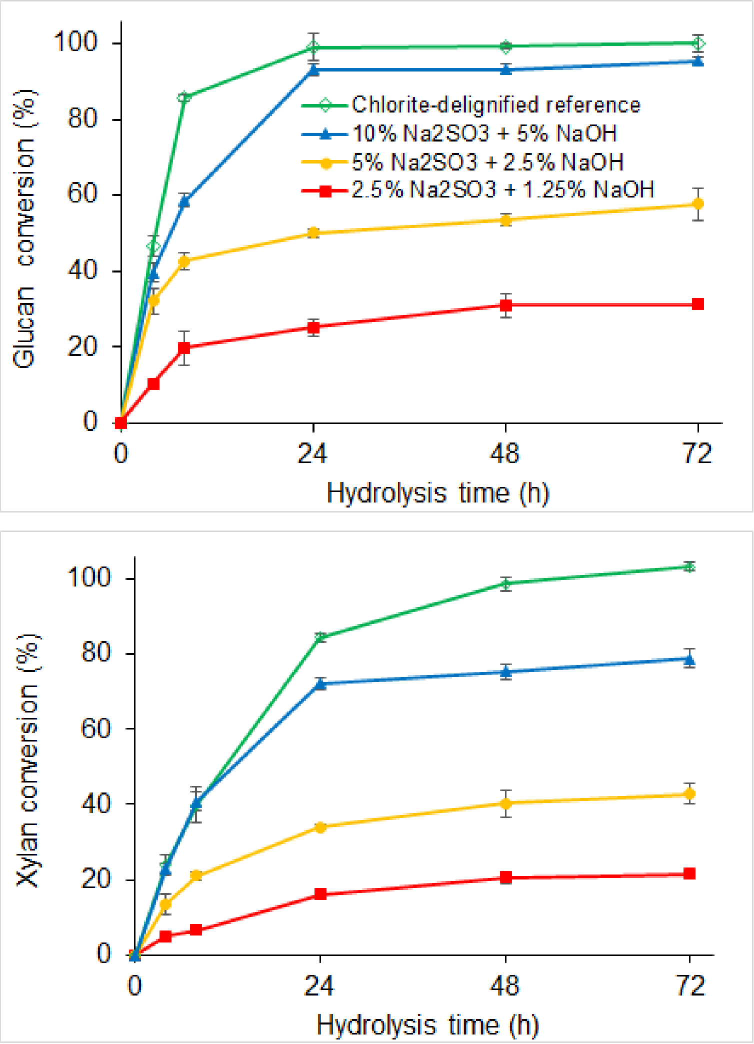

Figure S1: Time course of the enzymatic glucan and xylan conversion of sugarcane bagasse after chemithermomechanical pretreatment with increasing alkaline-sulfite loads. A reference chlorite-delignified sugarcane bagasse sample is also included in the dataset.
