# Supplementary material for: Fate of p-hydroxycinnamates and structural characteristics of residual hemicelluloses and lignin during alkaline-sulfite chemithermomechanical pretreatment of sugarcane bagasse
Source: Biotechnol Biofuels. 2018 Jun 5;11:153. doi: 10.1186/s13068-018-1155-3 (PMC5987574; doi:10.1186/s13068-018-1155-3)
Supplement: Supplementary file 2 — Additional file 2: Table S1. Assignments of 1H/13C correlation signals in the 2D HSQC spectra from untreated and pretreated sugarcane (Saccharum spp.) bagasse in DMSO-d6. [file 13068_2018_1155_MOESM2_ESM.pdf]

Table S1. Assignments of $^{1}H/^{13}C$ correlation signals in the 2D HSQC spectra from untreated and pretreated sugarcane (*Saccharum* spp.) bagasse in DMSO-d_6.

Label	δ_C/δ_H (ppm)	Assignment
Lignin cross-peak signals		
-OCH_3	55.6/3.73	C/H in methoxyls
A_γ	59.4 /3.40 and 3.72	C_γ/H_γ in normal (γ-hydroxylated) β–*O*–4´ substructures (**A**)
I_γ	61.3/4.09	C_γ/H_γ in cinnamyl alcohol end-groups (**I**)
A'_γ	62.7/3.83-4.30	C_γ/H_γ in γ-*p*-coumaroylated β–*O*–4´ substructures (**A'**)
A_α/A'_α	71.8/4.87	C_α/H_α in β–*O*–4´ substructures (**A, A'**)
$A'_{\beta(S)}$	83.0/4.33	C_β/H_β in γ- *p*-coumaroylated β–*O*–4´ substructures linked to a S unit (**A'**)
$A_{\beta(G)}$	83.6/4.29	C_β/H_β in β–*O*–4´ substructures linked to a G unit (**A**)
$A_{\beta(S)}$	85.9/4.12	C_β/H_β in β–*O*–4´ substructures linked to a S unit (**A**)
$S_{2,6}$	103.8/6.69	C_2/H_2 and C_6/H_6 in etherified syringyl units (**S**)
G_2	110.9/7.00	C_2/H_2 in guaiacyl units (**G**)
FA_2	111.0/7.32	C_2/H_2 in ferulic acid units (**FA**)
PCA_β and FA_β	113.5/6.27	C_β/H_β in *p*-coumarates (**PCA**) and ferulates (**FA**)
G_5/G_6	114.9/6.72 and 6.94 118.7/6.77	C_5/H_5 and C_6/H_6 in guaiacyl units (**G**)
$PCA_{3,5}$	115.5/6.77	C_3/H_3 and C_5/H_5 in *p*-coumarates (**PCA**)
FA_6	123.3/7.10	C_6/H_6 in ferulates (**FA**)
$PCA_{2,6}$	130.0/7.46	C_2/H_2 and C_6/H_6 in *p*-coumarates (**PCA**)
PCA_α and FA_α	144.4/7.41	C_α/H_α in *p*-coumarates (**PCA**) and ferulates (**FA**)
Polysaccharide cross-peak signals		
X_5	63.2/3.26 and 3.95	C_5/H_5 in β-D-xylopyranoside
X_2	72.9/3.14	C_2/H_2 in β-D-xylopyranoside
X'_2	73.5/4.61	C_2/H_2 in 2-*O*-acetyl-β-D-xylopyranoside
X_3	74.1/3.32	C_3/H_3 in β-D-xylopyranoside
X'_3	74.9/4.91	C_3/H_3 in 3-*O*-acetyl-β-D-xylopyranoside
X_4	75.6/3.63	C_4/H_4 in β-D-xylopyranoside
U_4	81.4/3.22	C_4/H_4 in 4-*O*-methyl-α-D-glucuronic acid
U_1	97.7/5.32	C_1/H_1 in 4-*O*-methyl-α-D-glucuronic acid
X'_1	99.8/4.58	C_1/H_1 in 2-*O*-acetyl-β-D-xylopyranoside
X_1/X'_1	102.1/4.38	C_1/H_1 in β-D-xylopyranoside + 3-*O*-acetyl-β-D-xylopyranoside
Gl_1	103.3/4.41	C_1/H_1 in (1→4)-β-D-glucopyranoside
Gl_1	103.6/4.30	C_1/H_1 in (1→3)-β-D-glucopyranoside + (1→6)-β-D-glucopyranoside
