# Supplementary material for: Fate of p-hydroxycinnamates and structural characteristics of residual hemicelluloses and lignin during alkaline-sulfite chemithermomechanical pretreatment of sugarcane bagasse
Source: Biotechnol Biofuels. 2018 Jun 5;11:153. doi: 10.1186/s13068-018-1155-3 (PMC5987574; doi:10.1186/s13068-018-1155-3)
Supplement: Supplementary file 3 — Additional file 3: Figure S2. Processes diagram, main process steps and transformations, and mass balances for sugarcane bagasse and total pCAs and FAs by treatment and analytical procedure. The photomicrography of the untreated sugarcane bagasse was obtained after toluidine blue staining. [file 13068_2018_1155_MOESM3_ESM.pdf]

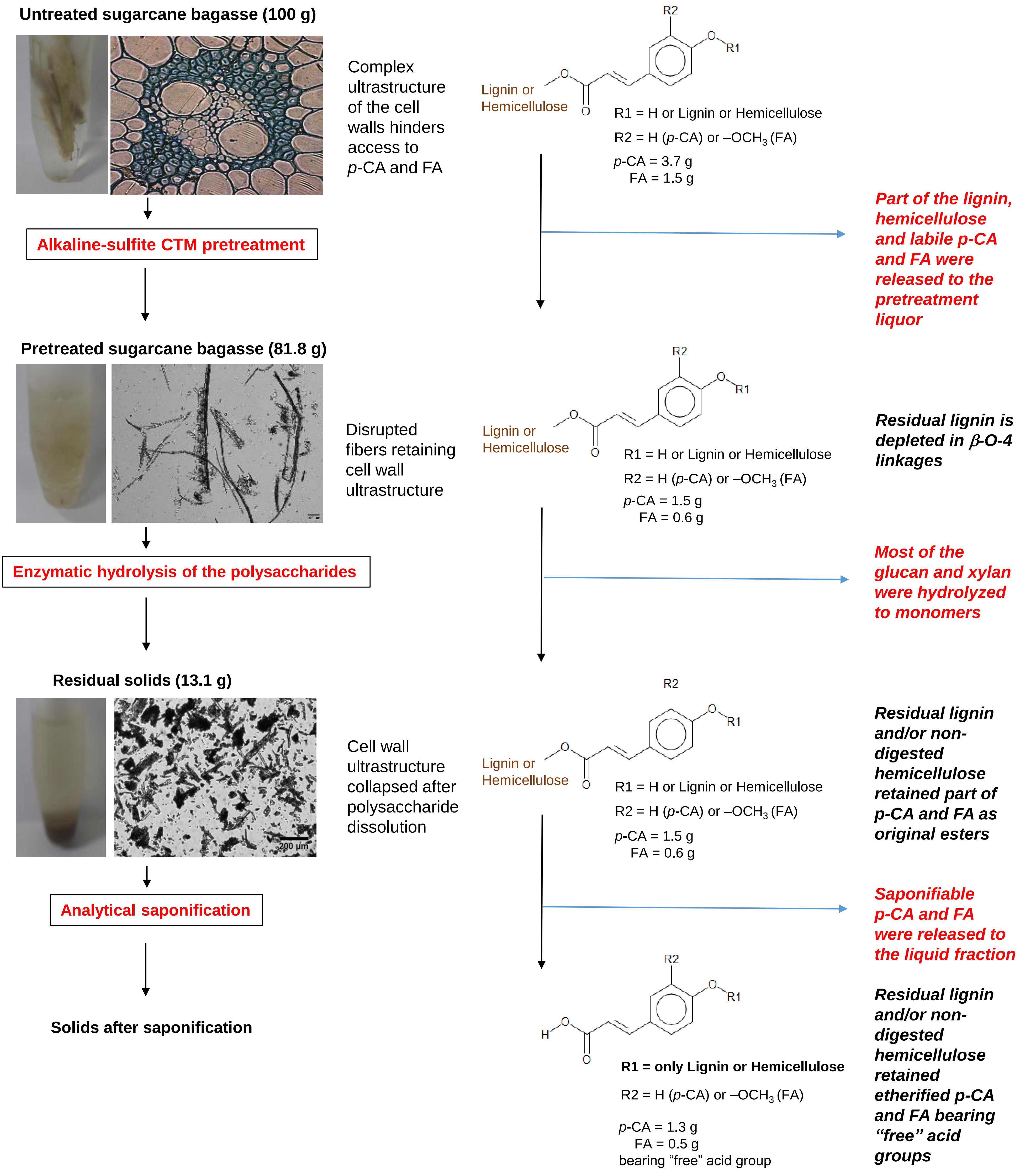

Figure S2. Processes diagram, main process steps and transformations, and mass balance for sugarcane bagasse and total *p*CA and FA along treatments and analytical procedures. The photomicrography from untreated sugarcane bagasse was obtained after toluidine blue staining.
